# Production of non-glycosylated recombinant Pla l 1, the main allergen of *Plantago lanceolata*

**DOI:** 10.1186/2045-7022-4-S2-P15

**Published:** 2014-03-17

**Authors:** Teresa Stemeseder, Stephanie Eichhorn, Sabrina Schuller, Roland Lang, Peter Briza, Thomas Hawranek, Fatima Ferreira, Gabriele Gadermaier

**Affiliations:** 1 University of Salzburg, Department of Molecular Biology, Division of Allergy and Immunology, Salzburg, Austria; 2 University of Salzburg, Christian Doppler Laboratory for Biosimilar Characterization, Salzburg, Austria; 3 Paracelsus Medical University Salzburg, Department of Dermatology, Salzburg, Austria

## Background

Pollen of Plantago lanceolata (English plantain) represent a frequent cause of allergic reactions in patients in the temperate regions of Europe, Australia and North America. The major allergen Pla l 1 is an Ole e 1-like protein and has been produced as glycosylated protein in yeast. The aim of the study was to express a non-glycosylated Pla l 1 and compare to the natural allergen.

## Methods

Recombinant Pla l 1.0101 was heterologously expressed in the E. coli strain Rosetta-gami B pLysS and purified using cation exchange and size exclusion chromatography. Natural Pla l 1 was obtained by pollen extraction and cation exchange chromatography. Physico-chemical properties of the purified proteins were analyzed in gel electrophoresis, mass spectrometry and circular dichroism. Using sera from Austrian ribwort pollen allergic patients (n=20) the IgE-binding activity of natural and recombinant Pla l 1 was investigated in ELISA.

## Results

Recombinant Pla l 1 was produced as non-tagged protein with a purity of > 95% and yielded 24 mg per liter of expression culture. The protein migrated at 15 kDa similar to the non-glycosylated natural counterpart. Mass spectrometry confirmed the primary sequence of rPla l 1 and the formation of three disulfide bonds. Natural Pla l 1 was purified as a mixture of non-glycosylated and glycosylated protein migrating at 15 and 17 kDa respectively. CD spectrometry revealed an unusual spectrum for the native protein with negative minima at 210 and 204 nm. Thermal denaturation resulted in unfolding of the protein which was only partially restored upon renaturation. Similar but lower IgE-reactivity of recombinant Pla l 1 compared to natural Pla l 1 was observed using patients' sera allergic to English plantain.

## Conclusion

The availability of non-glycosylated Pla l 1 facilitates molecule-based diagnosis of plantain allergy avoiding unspecific and clinically irrelevant reactivity due to carbohydrate moieties.

